# Mitochondrial dysfunction as a central hub linking Na^+^/Ca^2+^ homeostasis and inflammation in ischemic arrhythmias: therapeutic implications

**DOI:** 10.3389/fcvm.2025.1506501

**Published:** 2025-08-12

**Authors:** Siyu Sun, Zhanrui Zhang, Yuxi Li, Hui Zhang, Huige Guo, Guohui Chen, Pei Wei, Fei Lin, Guoan Zhao

**Affiliations:** Department of Cardiology, Life Science Center, The First Affiliated Hospital of Xinxiang Medical University, Weihui, Henan, China

**Keywords:** myocardial infarction, ventricular arrhythmia, mitochondrial, inflammation, Na^+^/Ca^2+^ homeostasis, fibrosis

## Abstract

Ventricular arrhythmia is the primary cause of sudden cardiac death in patients with myocardial infarction (MI). Myocardial inflammation and Na^+^/Ca^2+^ imbalance are the main triggering factors for life-threatening tachyarrhythmias after MI, which induce ion channel dysfunction, intracellular environment imbalance, tissue damage, and other alterations, subsequently resulting in modifications in cardiac conduction velocity and pathways. Subsequent adverse fibrotic remodeling provides a substrate for ventricular tachyarrhythmia (VT). Mitochondria, as the intersection site of these pathophysiological changes and the center of Na^+^/Ca^2+^ homeostasis and inflammatory crosstalk, may be key sites for the occurrence and development of ischemic arrhythmia. This review briefly outlines the roles of inflammation, Na^+^/Ca^2+^ homeostasis, and mitochondria in the damage, repair, and structural remodeling of infarcted hearts, in which these three are interconnected to provide a large number of substrates for VT.

## 1 Introduction

Ventricular arrhythmia (VA) is the main cause of sudden cardiac death (SCD) in patients with myocardial infarction (MI) (1). SCD accounts for approximately 50% of all cardiovascular deaths and is the primary manifestation of heart disease (2). Approximately 250,000–400,000 people die from SCD annually in the United States. In North America and Europe, the annual incidence of SCD in the general population ranges from 50 to 100 deaths per 100,000 individuals. In China, the incidence of SCD is approximately 41.84/100,000, which has significantly increased. While primary and secondary prevention has improved over recent years and the mortality rate from coronary heart disease has substantially decreased, the decline in SCD rate has been much smaller (3, 4). In addition to congenital cardiac structural abnormalities, cardiomyopathy, and primary cardiac ion channel diseases, slowing of conduction and increased dispersion of action potential repolarization caused by ischemia play important roles in SCD occurrence (5).

Myocardial inflammation and Na^+^/Ca^2+^ imbalance are the main triggering factors of life-threatening tachyarrhythmias after MI (6, 7). In the early stages, the aggregation of inflammatory factors mediates myocardial cell damage, leading to a change in ion channel function and a direct action on arrhythmia. In the subsequent healing phase of MI, inflammatory cells not only activate the repair of myofibroblasts and vascular cells but may also cause adverse fibrotic remodeling of the living segment. Cardiac hemodynamic and structural changes can cause left ventricular dilatation and dysfunction, providing a substrate for the ventricular tachyarrhythmia (VT) reentry circuit (8). With these structural modifications, alterations in intercellular coupling and ion channels further augment the susceptibility to VA. Therefore, the inhibition of myocardial inflammation, electrophysiological changes in the Na^+^/Ca^2+^ imbalance, and subsequent structural changes can significantly prevent the occurrence of VA and reduce SCD mortality in patients with MI.

Mitochondria are the main organelles of cardiomyocytes and are primarily responsible for adenosine triphosphate (ATP) production, metabolic regulation, oxidative stress, and inflammatory responses (9). In recent record, abnormal automaticity, triggered activity, and reentry are the three main mechanisms underlying cardiac arrhythmia (10). Mitochondrial dysfunction is closely associated with cardiac arrhythmia. For instance, triggered activity is caused by diastolic sarcoplasmic reticulum (SR) Ca^2+^ release. The mitochondrial Ca^2+^ content affects SR Ca^2+^ release by activating the ryanodine receptor 2 (RyR2) channels (11). Mitochondrial dysfunction causes abnormal ion channel function, Na^+^/Ca^2+^ imbalance, increased reactive oxygen species (ROS) production, changes in mitochondrial permeability, and activation of inflammatory factors, which in turn cause apoptosis and lead to fibrosis (12, 13). Fibrosis is one of the substrates for reentry (14). This review briefly outlines the roles of inflammation, Na^+^/Ca^2+^ homeostasis, and mitochondria in the damage, repair, and structural remodeling of the infarcted heart; describes how these three are interconnected in this dynamic process to provide a large number of substrates for VA; and discusses the difficulties and challenges faced by current related research and clinical practice.

## 2 Mechanism of ischemic arrhythmias

Electrophysiological changes in the ischemic region after acute MI are rapid, ranging from normal electrical activation to severe abnormal electrical activation, and repolarization occurs within 1 min. In the early stages of MI, the action potential duration (APD) is shortened, the amplitude is decreased, and the ascending velocity of the ascending branch is slowed, followed by a significant post-repolarization refractory period. The excitation threshold decreases at 1–3 min after coronary artery occlusion and then increases rapidly. At approximately 5 min after coronary artery occlusion, the threshold is 10 times higher than that before coronary artery occlusion, and excitability progressively increases as the tissue transitions from the normal area to the ischemic area. The absolute and relative refractory periods of the ischemic myocardium are shortened by 40–50 ms. At approximately 15 min after coronary occlusion, the cells completely lose their ability to respond, and their excitability gradually disappears. Coronary artery occlusion occurs at 20 min to 2 h after the occurrence of conduction disorders, along with prolonged mild simple conduction time and severe complete atrioventricular block, resulting in arrhythmia. Electrocardiography reveals ST segment elevation, delayed activation, QRS fragmentation, t-waves and QRS alternans, and conduction block (15).

### 2.1 Altered functions of multiple ion channels

Changes in ion channel function play an important role in the electrophysiological changes that occur during arrhythmias. The dysfunction of various cardiac ion channels, such as Na^+^, K^+^, and Ca^2+^ channels, increases the susceptibility to arrhythmia after MI (16, 17). K^+^ starts to change during the early stages of acute myocardial ischemia. Long-term ischemia leads to an increase in extracellular K^+^ concentration, which is the substrate and trigger for cardiac conduction velocity (CV) changes and arrhythmia (18). An increase in extracellular K^+^ concentration changes the resting membrane potential of cardiomyocytes, reduce the activity of voltage-gated Na^+^ channels, leads to a decrease in cell excitability and CV, and promotes unidirectional blockage and reentry (19, 20). CV and APD are important factors in the occurrence of arrhythmias, and changes in CV play important roles in the generation and maintenance of arrhythmias. Voltage-optical mapping studies of isolated hearts have shown that the induction of ventricular fibrillation (VF) at high activation frequencies is associated with decreased CV (21). It has also been suggested that arrhythmias may result from local CV heterogeneity (22). Many factors affect the CV, including coupling with non-muscle cells (23, 24), extracellular gaps, gap links, and extracellular ion concentrations (25–27). Among these, the upstroke velocity of the action potential is the key factor that affects CV, and the upstroke velocity mainly depends on the recovery of the Na^+^ channel. Therefore, the CV is closely related to Na^+^ channels (28).

The major subtype of voltage-gated Na^+^ channels, Na_v_1.5, encoded by the *SCN5A* gene, is mainly expressed in the intercalated discs of the heart. It is the key channel for maintaining a normal CV and determining the excitability and conductivity of the heart. It also interacts with cAMP-dependent protein kinase A (PKA) and calmodulin-dependent kinase II (CaMKII). It can bind to various proteins such as CaMKII and membrane-associated guanylate kinase (MAGUK) to form macromolecular compounds that regulate gene transcription, protein synthesis, trafficking, membrane incorporation, channel function, and ultimately degradation (29, 30). Na_v_1.5 remodeling is a key basis for the occurrence of VA reentry into the border zone of MI. Recent studies have shown that the autoimmune response against Na_v_1.5 can cause conduction defects (31) and complete inactivation of Na_v_1.5 due to a molecular dynamics disorder, can cause long QT syndrome type 3 (LQT3). Non-equilibrium gating leading to decreased availability of the Na_v_1.5 closed conformation can cause Brugada syndrome (BrS). Moreover, Na_v_1.5, dysfunction can lead to VA in pathophysiological conditions of heart disease, such as heart failure, dilated cardiomyopathy, and diabetic heart disease (32–34). Previous studies have determined that *SCN5A* mutations can induce a decrease in the amount and function of Na_v_1.5, using genetic, electrophysiological, and molecular methods, leading to a series of VA such as LQT3, BrS, torsades de pointes, and idiopathic ventricular fibrillation. This process can be affected by time, temperature, environmental factors, and genetic factors (35, 36).

The heart beats rhythmically to drive the blood through the body. Cardiac action potentials are generated by the simultaneous opening and closing of many transmembrane ion channels. Dynamic changes in Ca^2+^ concentration play a key role in this process (37). RyR2 is the main Ca^2+^ release channel during systole and sarcoendoplasmic reticulum Ca^2+^-ATPase (SERCA) is the main Ca^2+^ uptake channel during systole in the SR, which is involved in excitation-contraction coupling. The amount of Ca^2+^ released by the SR through RyR2 largely determines the Ca^2+^ transient state (38). Ca^2+^ flows into the cell and is released from the SR via RyR2 to trigger the contractile myocardium, after which Ca^2+^ is mostly taken up or loaded into the SR via SERCA to trigger the diastolic myocardium. Genetic and acquired defects in RyR2 or SERCA have been suggested to be associated with a range of heart diseases, including life-threatening arrhythmias and heart failure (39) (40). These defects typically manifest as an impaired ability of RyR2 to remain closed during the diastolic phase of the cardiac cycle, resulting in enhanced diastolic Ca^2+^ release (DCR, manifested as Ca^2+^ sparks and Ca^2+^ waves) (41). The diastolic release of Ca^2+^ from the SR leads to prolonged APD and increased arrhythmic risk (10). Increasing the RyR2 activity in the ventricle and alleviating its inhibitory effect on RyR2-mediated Ca^2+^ release have been reported to independently cause catecholaminergic polymorphic VT (42). Furthermore, Xie et al. discovered that type 2a *SERCA* (*SERCA2a*) knockdown mice had a reduced arrhythmic risk of ischemic cardiomyopathy due to decreased SR diastolic Ca^2+^ leak (40). Therefore, intracellular Ca^2+^ homeostasis imbalance is a key factor in ischemic arrhythmia (43).

CaMKII is a multifunctional serine/threonine protein kinase widely expressed *in vivo*. Its activity is mainly modified and regulated by changes in intracellular Ca^2+^ content, and regulates intracellular Ca^2+^ dynamics, contractility, metabolism, and gene expression by phosphorylating various downstream targets (44). CaMKII has been identified as an important modulator of excitation-contraction and excitation-transcription coupling, a key determinant of the response to pathological cardiac remodeling, and is activated upon MI. CaMKII exerts proarrhythmic signaling through a large number of ion channels and SR-related proteins. CaMKII is known to activate L-type calcium channels, various K^+^ channels, Na_v_1.5 and Na_v_1.8 (45, 46). Stimulation of these channels results in early and delayed depolarization and spatially dispersed increases in repolarization, which promote arrhythmias, such as atrial fibrillation, ventricular tachycardia, and VF. CaMKII can also phosphorylate RyR2 and promote Ca^2+^ release from the SR into the cytoplasm (47). This SR Ca^2+^ leakage can activate proarrhythmic Ca^2+^-sensitive conductance. Overexpression of CaMKII has also been shown to induce structural and electrical remodeling of the heart, leading to impaired contractility and an increased risk of SCD. Conversely, inhibition of CaMKII helps maintain intracellular Ca^2+^ homeostasis after pressure overload and ischemic stress to prevent adverse electrical remodeling after MI (48, 49). Previous studies have demonstrated that CaMKII co-immunoprecipitates with Na_v_1.5, and experiments have shown a stable physical interaction between phosphorylated CaMKIIδC and L1 of Na_v_1.5. Phosphorylation of CaMKII enhances the inhibition of the late depolarization current of Na_v_1.5, leading to the prolongation of the action potential, further disrupting Ca^2+^ homeostasis, and providing additional substrates for arrhythmia formation (50).

### 2.2 Inflammation

Inflammation is thought to trigger arrhythmia following MI. In an analysis of 478,524 individuals from the UK Biobank cohort, C-reactive protein (CRP) levels were found to be significantly and positively associated with the risk of developing atrial fibrillation. The heart rate for atrial fibrillation events increased significantly with increasing neutrophil count, monocyte count, and neutrophil-to-lymphocyte ratio (NLR), whereas the levels of systemic infection markers had an even stronger relationship with VA risk than the levels of systemic infection markers with atrial fibrillation risk. Restricted cubic spline analysis of the fully adjusted model showed that the risk of developing VA increased monotonically with increasing CRP levels and neutrophil counts; a similar association was observed between monocyte count, NLR, and VA occurrence (51). It adds precipitants and substrates to the VA in both the early acute injury phase and the subsequent chronic repair phase.

Repair of the infarcted heart depends on the timely suppression of the inflammatory response and the resolution of inflammatory infiltration after infarction. Damage-associated molecular pattern proteins released by necrotic cells after early MI trigger local and systemic inflammatory responses. Various inflammatory factors directly induce arrhythmias and recruit large numbers of neutrophils and monocytes. Under the action of inflammatory factors, recruited white blood cells change the function of ion channels and cause membrane potential changes (52). Simultaneously, many white blood cells infiltrate and exchange ions, nucleotides, metabolites, and electrical signals with the cardiomyocytes via connexins. The quantitative change and redistribution of connexins leads to gap junction remodeling, which is an important factor in inducing arrhythmia (53). Macrophages couple with cardiomyocytes through gap junctions containing connexin 43(Cx43), undergo synchronous depolarization, and participate in normal and abnormal cardiac conduction. Moreover, computer simulations have shown that an increased number of such junctions reduces the action potential upshoot and overshoot, leading to earlier repolarization and a shorter refractory period (54).

In addition, fever, increased heart rate, and increased oxygen consumption caused by a systemic inflammatory response can further promote arrhythmia and expansion of the lesion area. Subsequently, white blood cells remove dead cells and matrix debris through phagocytosis, thereby providing an environment for subsequent repair of the infarct area. An appropriate early inflammatory response can reduce the infarct area, promote scar formation, maintain the stability of the environment in the peri-infarct area, and contribute to recovery of the ischemic myocardium. Excessive and prolonged inflammatory responses can lead to apoptosis of myocardial cells, hypertrophy, and fibrosis of myocardial tissue in the non-infarcted area, leading to adverse remodeling of ischemia-related tissues and myocardial electrophysiological dysfunction. Monocyte and macrophage subsets secrete cytokines and growth factors that coordinate the repair, recruitment, and activation of mesenchymal cells, such as cardiac fibroblasts and vascular endothelial cells (55). Activated mesenchymal cells secrete a large number of extracellular matrix proteins (56), promote the formation of myocardial fibrosis scars, and thus provide a matrix for reentry, which is closely related to arrhythmia (57).

### 2.3 Increased fibrosis

The heart adaptively responds to pathological injury, leading to cardiac remodeling characterized by cardiomyocyte hypertrophy and fibrosis. This remodeling includes chronic remodeling under volume/pressure load and acute repair under ischemia-hypoxia injury. During MI, phagocytosis of necrotic cells and tissues activates anti-inflammatory pathways that inhibit cytokine and chemokine signaling (58). Activation of the renin-angiotensin-aldosterone system and release of transforming growth factor-β induce the transformation of fibroblasts into myofibroblasts, causing ventricular fibrosis, similar to the macrophages mentioned above. When the number of myofibrocytes is considerable, nonmyocardial cells in the heart become obstacles to the propagation of action potentials. The differentiation state of fibroblasts has been previously shown to be associated with a change in the expression profile of ion channels, and the transition from fibroblasts to myofibroblasts can increase Na_v_1.5; furthermore, they display rapid inward voltage-gated sodium currents that exhibit biophysical properties similar to the sodium currents found in cardiomyocytes (59). *in vitro*-cultured fibroblasts can electrically couple with cardiomyocytes to participate in excitation conduction through Cx43, and these intercellular connections allow myofibroblasts to influence the electrical activity of cardiomyocytes (60). Previous studies have generated mice that specifically express the optogenetic cation channel *ChR2*(*H134R*) in cardiac fibroblasts. After MI, fibroblasts are highly expressed in the injured area and close to cardiomyocytes in scar tissue, and light stimulation of the scar tissue can cause excitation of the whole heart and induce arrhythmia. Cx43 and other gap junction proteins, which are thought to mediate the coupling between cardiomyocytes and fibroblasts, are not required. Gap junctions and ephaptic coupling mediate the coupling between cardiomyocytes and fibroblasts in a cooperative but functionally redundant manner; however, this fibroblast-muscle coupling is not as strong as myofibroblast-myocyte coupling (61). These results suggest that electrical coupling between myofibroblasts and cardiomyocytes can destroy the original electrophysiological activity of the myocardium, induce electrophysiological abnormalities such as ectopic automaticity, posterior depolarization, and reentry, and promote the occurrence and development of arrhythmia.

In addition to directly affecting the electrical activity by coupling with cardiomyocytes, fibroblasts, which are the main cells producing extracellular matrix, are activated in large numbers after MI, promoting the deposition of extracellular matrix proteins (62). Various extracellular matrix proteins bind to cytokines, growth factors, and cell surface receptors to regulate the cell phenotype, thereby indirectly affecting arrhythmia. Structural remodeling of atrial fibrillation has been reported to involve the accumulation of cross-linked collagen in atrial fibroblasts. In a previous report, calcitonin receptor-knockout mice exhibited atrial fibrosis and increased susceptibility to atrial fibrillation due to collagen accumulation (63). A large amount of collagen and extracellular matrix can mechanically separate cardiomyocytes, destroy the continuity of myocardial bundles, interfere with the gap junction of cardiomyocytes, destroy the electrical coupling between cardiomyocytes, and cause discontinuous or “zigzag” conduction, leading to slow CV, unidirectional conduction block, and prolongation of the conduction path between cardiomyocytes, thus inducing arrhythmia. Myocardial fibrosis is an essential substrate for arrhythmias (64).

After MI, three distinct structural regions emerge in the left ventricle: the infarct, transition boundary, and remote zones (65). Magnetic resonance imaging shows that a large peri-infarct transition border zone is the single factor in the inducibility of monomorphic VT, providing mechanistic support for the association between peri-infarct size and mortality. Tissue inhomogeneity in the infarct border may provide a substrate for underlying reentrant arrhythmias, leading to SCD (66). In a study of 686 patients with apparent idiopathic nonsustained VA, left ventricular scars with annular patterns were associated with malignant arrhythmic events on cardiac magnetic resonance imaging. All patients with annular scars showed VA with a right bundle branch block, and multifocal VA was observed in 46% of patients. The prevalence of multifocal VA is much higher in patients with annular scars than in those with non-annular scars, suggesting that the influence of a specific infarct shape on VA should not be ignored (67). In addition, other studies have shown that the effect of fibrotic areas <20% and >80% on arrhythmia is relatively benign and that the arrhythmogenic effect is usually maximal at 30%–50% of the fibrotic area (64). Thus, inhibiting the progression of fibrosis without affecting MI healing may reduce the risk of arrhythmias.

### 2.4 Crosstalk among Na^+^/Ca^2+^ homeostasis, inflammation, and fibrosis

In most pathological conditions, arrhythmia is often accompanied by inflammation and structural and electrical remodeling, with crosstalk. Upon initial ischemic injury, monocytes and macrophages are recruited and polarized to a proinflammatory phenotype, secreting inflammatory factors (such as IL-6, IL-1, and TNF-α), triggering a cytokine cascade. Inflammation rapidly induces the effect of cytokines on the expression of ion channels, which directly prolongs the QTc interval, and changes in these ion channels are negatively correlated with changes in CRP and IL-1 in patients. Although these changes are transient, they may significantly increase the risk of developing life-threatening VA in these patients (68). In addition, cardiac fibroblasts respond to IL-1 by acquiring a proinflammatory and matrix-degrading phenotype, delaying myofibroblast transformation and preventing premature acquisition of a matrix-synthetic phenotype until the infarct clears dead cells and matrix debris (69). In addition, some members of the chemokine family may also affect non-hematopoietic cells, such as cardiomyocytes, fibroblasts, and vascular cells, which mediate the transformation of fibroblasts into myofibroblasts or the recruitment of monocytes and neutrophils with fibroblast characteristics to promote the development of fibrosis (70, 71), which increases the axial resistance of the sarcoplasm. This enhances the coupling between fibroblasts and cardiomyocytes, both of which reduce the CV and increase the CV dispersion. As the activation of pro-inflammatory signals leads to cardiomyocyte death, mitochondrial membrane permeability transition pores open, perturb the intracellular Ca^2+^ balance, and increase ROS, triggering arrhythmic events. When intracellular Ca^2+^ increases in cardiomyocytes, CaMKII phosphorylation increases and activates IκB kinase, nuclear factor kappa B (NF-κB) is deinhibited, macrophage infiltration in the ischemic area increases, fibrosis scars become larger, and cardiac function is weakened (72). In the absence of endogenous CaMKII inhibitor 1 (CaMK2n1), the increased activation of CaMKIIδ-p38/JNK-NLRP3 inflammasome pathway leads to aggravated cardiomyocyte inflammation, aggravated ventricular remodeling and malignant VA (73). Single-cell sequencing of the infarcted and non-infarcted regions of ischemic cardiomyopathy revealed a large amount of leukocyte infiltration in the fibrotic myocardium, especially of proinflammatory CD^4+^ T cells (74). The presence of these inflammatory cells suggests that myofibroblast apoptosis occurs during the transition from the proliferative to mature phase of healing in the infarcted area, which may be regulated by inflammation (75). The increase in fibrosis can cause partial uncoupling of muscle fibers, a zigzag path of wave conduction, and slow or blocked conduction, which eventually leads to the occurrence of arrhythmia (76). Therefore, ischemic arrhythmia results from the crosstalk between the ion channel-fibrosis-inflammatory response and other factors.

## 3 The role of mitochondrial function as a central cross-linking point in ischemic arrhythmia

Mitochondria are widely distributed in cardiomyocytes, accounting for 30% of the total volume of adult cardiomyocytes (77). They produce ATP and regulate metabolism, oxidative stress, and inflammatory responses, which are common pathological changes in ischemic arrhythmia (9). Mitochondria also sense intracellular Ca^2+^ signals, mediate energy production and cell death (78), and play important roles in Ca^2+^ homeostasis in cardiomyocytes (79). Under normal conditions, fatty acids are the preferred energy substrates for ATP production in the myocardium. Fatty acids undergo β-oxidation in the mitochondria to produce acetyl-CoA, which enters the tricarboxylic acid cycle to produce ATP for energy (80).

### 3.1 Reduced mitochondrial function can cause mitochondrial Ca^2+^ overload in ischemic state

When the body undergoes hypoxia-ischemia, the mitochondrial metabolism changes from oxidative phosphorylation to glycolysis, which reduces oxygen consumption and ensures ATP output (81). This metabolic transition leads to an increase in lactic acid, a decrease in intracellular pH, and an increase in the concentration of H^+^ in both the intercellular space and within the cell (82). H^+^ is exchanged with Na^+^ through the Na^+^/H^+^ exchanger, resulting in an increase in intracellular Na^+^ concentration. Simultaneously, owing to the decrease in Na^+^/ K ^+^ -ATPase activity, the extracellular transport of Na^+^ is reduced, leading to its accumulation in cells. In addition, the influx of Na^+^ ions into cells through nonselective cation channels activated by membrane stretching also leads to an increase in intracellular Na^+^ concentration. This increase in Na^+^ further activates the sodium-calcium exchanger (NCX) to operate in a “reverse mode,” eventually leading to intracellular Ca^2+^ overload (83) and causing early afterdepolarization or delayed afterdepolarization. When multiple depolarization events reach the threshold for sodium channel activation, a series of tachyarrhythmias can be induced. If this is extremely insufficient to induce an action potential, it may exacerbate regional differences in repolarization, leading to alternating or unidirectional conduction blocks and reentry (84). When Ca^2+^ overload occurs, a large amount of Ca^2+^ enters mitochondria through the mitochondrial Ca^2+^ uniporter (MCU) complex (85). Ultimately, this results in mitochondrial Ca^2+^ overload.

### 3.2 Crosstalk between mitochondrial ROS and Na^+^/Ca^2+^ homeostasis

When mitochondrial calcium levels increase, the activity of the electron transport chain is stimulated, leading to a higher ROS release. Overproduction of mitochondrial-derived ROS may lead to the oxidation of RyR2 and further leakage of endoplasmic reticulum Ca^2+^, forming a vicious cycle (86). ROS also increases CaMKII phosphorylation. In ischemic heart disease, ROS production is increased in the infarct border region, which overactivates CaMKII phosphorylation and reduces INa density, thereby slowing the recovery rate after Na_v_1.5 inactivation. In addition, the delay in repolarization and prolongation of the effective refractory period lead to a decrease in cardiac CV and even a conduction block (87). Mitochondria are the central hub for immune system activation, and their dysfunction leads to many inflammatory diseases. On the one hand, it relies on ROS to trigger an inflammatory response. Impaired mitochondrial function leads to activation of the tricarboxylic acid cycle and an increase in nitric oxide synthase, which eventually causes an increase in ROS. However, this does not depend on ROS for triggering inflammatory responses.

### 3.3 Crosstalk among mitochondrial permeability transition pore and Na^+^/Ca^2+^ homeostasis and mROS

Mitochondrial Ca^2+^ binds to oxidized cardiolipin and triggers the release of the membrane gap protein cytochrome c into the cytoplasm. At the same time, the increase in Ca^2+^ and ROS levels can further open the mitochondrial permeability transition pore, leading to the immediate collapse of the mitochondrial membrane potential, release of cytochrome c, and activation of caspase protease, resulting in cardiomyocyte apoptosis (88). In the process, myocardial cells produce inflammatory factors such as TNF-α, IL-1β, and IL-6, leading to inflammatory response or dysfunction when myocardial injury occurs (89).

In recent years, studies have shown that, apart from the above effects through metabolism, inflammation, and oxidative stress, mitochondria are directly related to cell membrane Na_v_1.5 in structure and function, and this association is different in various mitochondrial subsets. Subsarcolemmal mitochondria are more closely related to Na_v_1.5 than to interfibrillar mitochondria and perinuclear subdomain mitochondria. This link may be established through the NCX, and its functional crosstalk includes sodium currents, Ca^2+^ dynamics, transcriptomics, and oxidative stress. Interestingly, the transcriptomics results showed that the negative correlation between *SCN5A* and *SCL8B1* may be a compensatory result, indicating that the functions of the mitochondria and sodium channels are complementary. However, the mechanism by which Na^+^ channel expression or activity affects mitochondrial function or integrity requires further studied (90).

## 4 Limitations

Mitochondrial research currently faces several challenges and limitations in the study of arrhythmias. In different regions of the heart and even within individual myocardial cells, there is significant functional heterogeneity in mitochondria. Traditional batch detection methods (such as western blotting and PCR) mask this heterogeneity. Single-cell sequencing and spatial transcriptomics have partially addressed this issue; however, they have limitations in mitochondria-specific analysis. Most existing studies focus on the overall mitochondrial function changes in the entire myocardial cell but neglect the precise spatial localization of mitochondria within the cells and the interactions between organelles. Bidirectional communication between the mitochondria and the T tubules, SR, and endoplasmic reticulum is accomplished through mitochondria-associated endoplasmic reticulum membranes, forming a complex structure. Disruption of this specific spatial relationship not only leads to calcium regulation imbalance but also causes dysregulation of multicellular organelles, becoming a triggering factor for arrhythmia. Nevertheless, the details of this spatially specific regulatory mechanism have not yet been fully elucidated (91, 92). In addition, there is real-time coupling between the cardiomyocyte energy status and ion channel function, and this metabolic-electrical coupling occurs rapidly and precisely (93). However, owing to the lack of dynamic detection technology, there is a lack of research on the simultaneous monitoring of metabolic changes and electrical activity at the millisecond level. This has led to a fundamental gap in our understanding of how metabolic alterations translate into electrical instability, with most conclusions remaining at the correlation level.

## 5 Future directions

Despite these difficulties, the potential use of mitochondria in the treatment of arrhythmias remains promising. The therapeutic implications of targeting mitochondria are anticipated. For example, the coenzyme Q10(antioxidants protect mitochondria and transfer electrons to facilitate energy metabolism) that is currently widely used in clinical practice has demonstrated the potential for anti-arrhythmic effects. Studies have shown that it can reduce the incidence of atrial fibrillation. Supplementation of mitochondria-targeted antioxidants such as mitoquinone can reduce mitochondrial membrane damage, maintain Na^+^/K^+^-ATPase activity, and reduce NCX-mediated Ca²^+^ influx. It is particularly applicable to arrhythmias related to mitochondrial oxidative stress (such as ischemia-reperfusion injury and metabolic cardiomyopathy), and has achieved good clinical evidence. Targeted MCU inhibitors, such as Ru360, reduce mitochondrial calcium overload, whereas the activation of NCX promoted Ca²^+^ efflux and restores intracellular calcium homeostasis in cardiomyocytes. The activation of the TLR4/NF-κB pathway can be suppressed by improving mitochondrial membrane permeability, such as using cyclosporine A to inhibit the opening of the mitochondrial permeability transition pore and reducing the release of mitochondrial DNA and ROS, thereby alleviating the inflammatory response. Peptide inhibitors have been used to selectively block the pathological mitochondrial fission protein Drp1 to prevent excessive mitochondrial fission in the early stages of mitochondrial injury and to reduce cardiomyocyte apoptosis (94).

For arrhythmias caused by specific mitochondrial DNA mutations or nuclear DNA mutations, gene therapy is at the forefront of exploration. Normal genes are introduced into the lesion model by using adeno-associated virus for gene replacement therapy. Another strategy is heterologous expression, where mitochondrial genes are re-encoded and then introduced into the cell nucleus, allowing them to be synthesized in the cytoplasm and targeted into mitochondria to compensate for the function of the mutated genes. Although gene therapy is not yet mature, it represents an important direction for the future.

In addition, Recent research has indicated that injecting healthy donor mitochondria into the induced pluripotent stem cells of patients with Barth syndrome can promote mitochondrial autophagy and biogenesis, improve mitochondrial respiratory function, and reduce the APD and frequency of arrhythmia (95). Although the existence of mitochondrial transplantation is relatively short-lived, this exciting discovery provides new directions for future research. In the future, we can differentiate high-purity cardiomyocytes from the induced pluripotent stem cells of patients themselves or immunologically matched donors, extract healthy mitochondria, and achieve efficient delivery through microinjection, nanotube-mediated delivery, or mitochondrial-targeting vectors (such as MITO-Porter). Thus, we can overcome the limitations of traditional drug thinking and develop mitochondrial replacement therapies. Although there are many studies on the role of mitochondria in arrhythmias, those that are actually used in clinical practice are relatively few. How to achieve clinical transformation in the future is also a major issue, and more clinical trials are needed for exploration.

## 6 Conclusion

**Figure 1:**
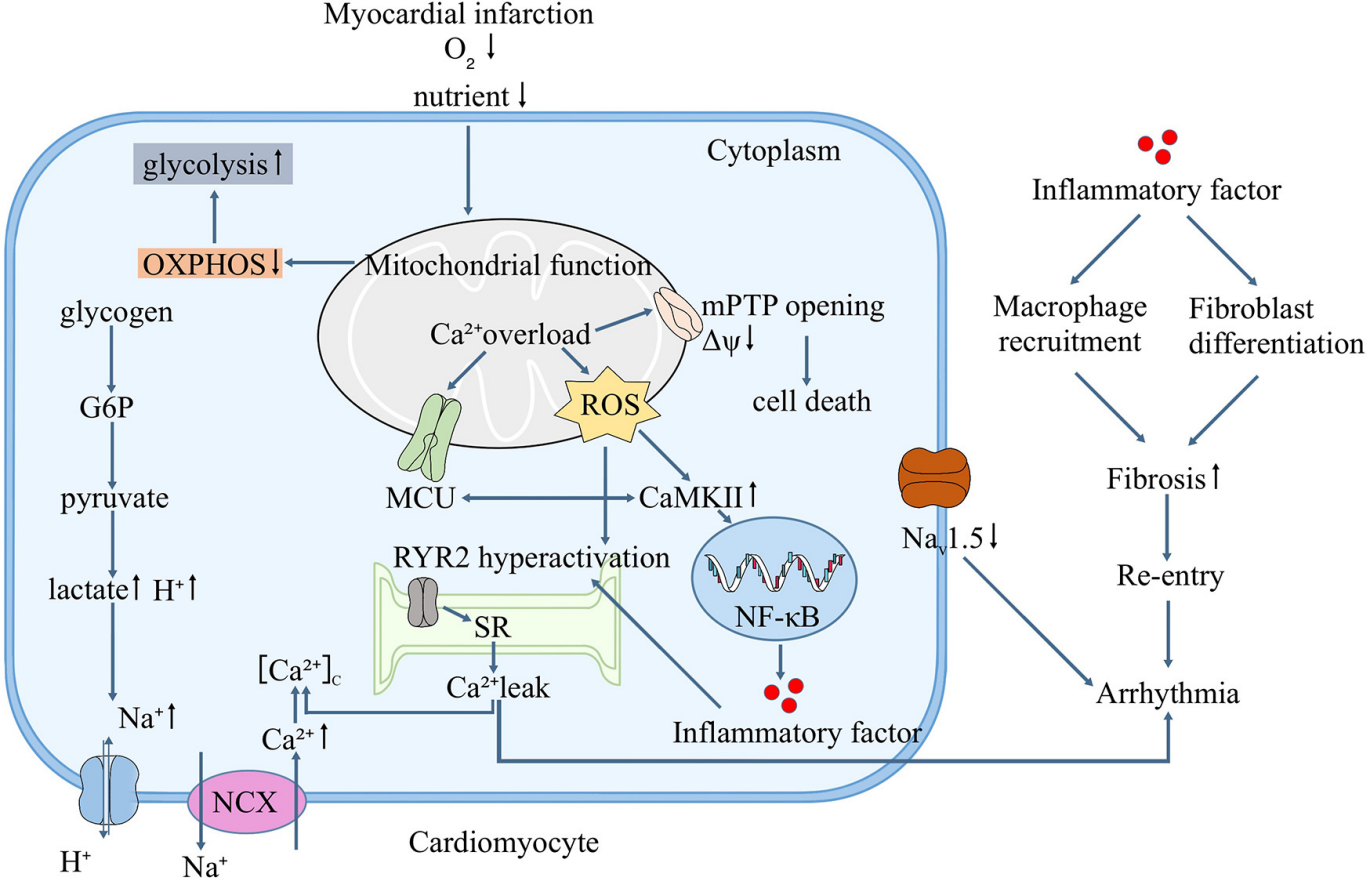
Mitochondrial function and the mechanism of ischemic arrhythmia.

In summary, the mitochondria, as a central site that regulates various cellular functions, are an important factor contributing to ischemic arrhythmia, which is a key point in the crosstalk among inflammation, Na^+^/Ca^2+^ homeostasis, and mitochondria and is also a potential therapeutic target. The specific mechanism is illustrated in Figure 1. Targeting the mitochondria to improve or restore function has been a popular topic in the treatment of ischemic and metabolic cardiomyopathies (96). However, because different etiologies of the disease produce different responses (97), preclinical data and clinical studies on such therapies remain insufficient. Herein, we reviewed and analyzed the role of mitochondria in the development of arrhythmia. If the mechanism between mitochondria and arrhythmia can be further clarified in the future, it may provide a new direction different from that of traditional arrhythmia treatment.
